# Development and Implementation of Baseline Welfare Assessment Protocol for Captive Breeding of Wild Ungulate—Punjab Urial (*Ovis vignei punjabiensis*, Lydekker 1913)

**DOI:** 10.3390/ani9121102

**Published:** 2019-12-09

**Authors:** Romaan Hayat Khattak, Zhensheng Liu, Liwei Teng

**Affiliations:** College of Wildlife and Protected Area, Northeast Forestry University, Harbin 150040, China; romaanktk@gmail.com (R.H.K.); tenglw1975@163.com (L.T.)

**Keywords:** welfare, protocol, veterinary assessment, Punjab urial, body condition, behavior, Pakistan

## Abstract

**Simple Summary:**

Current issues concerning animal welfare are receiving remarkable consideration among scientific communities and wildlife conservation organizations. In addition to the ethical and legal grounds of animal welfare, it is important to produce healthy and viable populations in captivity by ensuring optimum welfare. Wild species are reared in captivity but, unlike domestic animals, there is a lack of welfare assessment protocols for these wild species. In the current study, we developed and applied the first baseline welfare assessment protocol for Punjab urial (*Ovis vignei punjabiensis*). While developing this protocol, we gathered all possible and existing information about the biology and ecology of this species in its natural habitat. Since no welfare assessment protocol and husbandry guidelines exist for this species, we used a welfare assessment protocol for domestic sheep as the base and reference in developing the current protocol for captive Punjab urial. Later, we applied this protocol to three different herds of Punjab urial at two different facilities. Based on the initial results obtained, some areas were found to have shortcomings and recommended for quick corrective measures and improvements.

**Abstract:**

To ensure that captive breeding and other associated programs are more robust and sustainable, it is of utmost importance to ensure optimum welfare. Although it is well known that standard welfare is crucial for successful captive breeding, there is still a lack of welfare assessment protocols for wild species. The current study aimed to develop a leading baseline welfare assessment protocol for assessing welfare in captive Punjab urial. This protocol is based on the welfare protocol for domestic sheep from the Welfare Quality^®^ project, coupled with all the information obtained from the published literature on the species’ biology and ecology. This protocol consists of 4 principles, 12 criteria, and 31 animal- and resource-based indicators. The protocol was tested and applied to three different herds of Punjab urial at two different facilities. Initial results showed that some areas need to be improved for better captive breeding and management.

## 1. Introduction

To ensure that captive breeding programs are more robust and sustainable, it is important to ethically and legally ensure optimum animal welfare [1]. The rapid developmental processes of humans are affecting the natural habitats of wildlife. Thus, wildlife reservoirs, zoos, and enclosures must be adapted to minimize the effect of these changes [2]. Today, captive breeding is one of the most important conservation tools [3], providing an opportunity to the rare endangered species to produce stable populations for eventual release into the wild [4].

In the meantime, enclosures and other facilities for wild animals are under severe pressure to limit animals to small areas [5]. Thus, animal welfare subjects, particularly those related to the captive wild species, are rapidly recognized [6,7]. It is important to ensure the optimum levels of animal welfare of captive animals for the production and maintenance of healthy, viable populations [8]. To make captive breeding more robust, it is important to determine the main factors of species welfare and, most importantly, the welfare of every individual of a particular group [9].

Animal welfare assessment protocols can be designed by gathering information through simple inspections, animal observations, and visiting animal facilities and enclosures [10]. Animal welfare assessment protocols for livestock (poultry, cattle, and pigs) have already been developed under the auspices of the Welfare Quality^®^ project. These protocols are mainly based on animal-based indicators, in addition to also having resource or environmental measures [11]. Animal-based measures can be directly recorded by observing animals, including their physical appearance, health, and behavior. Unlike animal-based measures, environmental measures assess the available resources for these animals in captivity, and the animal itself is not taken into account. 

*Ovis orientalis* (urial) is a wild sheep that closely resembles Marco Polo sheep in general body appearance [12]. In Pakistan, the urial is represented by three subspecies: *Ovis vignei vigeni* (Ladakh urial), which is restricted to northern areas (Gilgit-Baltistan) of Pakistan; *Ovis vignei punjabiensis* (Punjab urial) found in the Salt Range (Punjab) and the northwestern province of Khyber Pakhtunkhwa (formerly known as the North-West Frontier Province); and *Ovis vignei blanfordi* (Baluchi urial), which is found in the southwestern province of Balochistan [13]. The Punjab urial is a gregarious ungulate, and most big herds include females, lambs, and immature males. It has been observed that the urial generally prefers grasses, but can also be found foraging on shrubs [14].

The species has been declared as vulnerable globally, with a declining population trend, according to the International Union for Conservation (IUCN) list of threatened species [15], and is endangered in Pakistan [16,17,18]. In Pakistan, wild ungulates are reared in captivity, but there are no standardized methods and protocols to measure the welfare of these captive ungulates. Therefore, this current study aimed to (i) design and develop a baseline welfare assessment protocol for captive Punjab urial based on the livestock welfare assessment protocol from the Welfare Quality^®^ project, (ii) implement this welfare assessment protocol in facilities hosting Punjab urial, and (iii) suggest recommendations for better captive breeding and management.

## 2. Materials and Methods 

For this study, we selected the subspecies *Ovis orientalis punjabiensis* because of its availability in captivity. The current study was conducted in two steps. In the first step, the welfare assessment protocol was developed, and, in the second step, the newly established protocol was implemented at captive facilities housing Punjab urial. We developed the welfare assessment protocol by combining results from other studies on the biology and behavior of the species and the sheep welfare assessment protocol [19], as both domestic sheep and Punjab urial belong to the family Bovidae.

To obtain information on the general biology and behavior of the species, we used Google Scholar and Web of Science search engines using “Ovis vignei” as keywords. Limited scientific published information is available regarding the biology of this species in natural habitats. Moreover, no work has been conducted to investigate the problems faced by this species in captivity. More than 31 scientific published papers were reviewed. Most of these published work focused on population status, population dynamics, diet ecology, and habitat, of which two papers [12,20] provided useful detailed information on the behavior and general biology of the species that was utilized for the welfare assessment protocol. 

For developing the Punjab urial welfare assessment protocol, four basic principles were taken into account: good feeding, good housing, good health, and suitable behavior [10,11]. These principles led to twelve criteria (see Section 3), which in turn allowed the development of welfare assessment indicators [21]. After combining information from [19,22], we developed an extensive set of 31 welfare indicators for Punjab urial (see Section 3). 

The final version of the welfare assessment protocol was then applied to three different groups of captive Punjab urial at two different facilities—Cherat Wildlife Park (CWP) in Nowshera and Manglot Wildlife Park (MWP) in Nizampur—in the month of August 2019. Both of these parks are located in the Nowshera District of Khyber Pakhtunkhwa Province, Pakistan. The captive breeding program was launched in 2008 at CWP with a single pair of founder animals, while in 2012 it was initiated at MWP, also with a single pair. The groups of both programs were mixed herds, including adult males, sub adult males, adult females, sub adult females, and lambs. CWP had one group consisting of 23 individuals (*n* = 23) with a mean age of 3.21 ± 2.21 years. At MWP, two groups were present (*n* = 6 and *n* = 8), with mean ages of 3.16 ± 1.57 and 3.33 ± 1.69 years, respectively. Groups were represented by coding their facilities (centers) as captive Punjab urial-1 (CU1) at CWP, and captive Punjab urial-2 (CU2) and captive Punjab urial-3 (CU3) at MWP. The protocol was applied by the same person. 

Statistical Analysis

In the current protocol, the observation time for social behavior was 120 min per herd in six 20-min sessions. Continuous focal sampling was used because these behaviors may be of short duration and elusive nature [10]. We performed the Shapiro–Wilk test with our datasets to check for normality. Later, data recorded for social behavior (*n* = number of occurrences of an event) for all three groups in the current protocol were analyzed using the nonparametric Kruskal–Wallis test to determine the significance of observed variables. All the statistical analyses were performed using SPSS version 23.0 (IBM Corp., Armonk, NY, USA).

## 3. Results

### 3.1. Development of Welfare Assessment Protocol 

The protocols developed in the current study for welfare assessment of Punjab urial consisted of 4 basic principles, 12 criteria, and 31 indicators (Table 1). Of the 12 criteria of the Welfare Quality^®^ protocol for domestic sheep, some were replaced keeping in view the biology of the species. The species under study is a wild ungulate, thus “lack of minerals” was added to the list of criteria. The current study is a leading step in captive Punjab urial welfare assessment, and will provide a base to investigate more complex and less frequently studied aspects in captive animals. 

#### 3.1.1. Lack of Prolonged Appetite 

Since there are no husbandry guidelines for this species, there is no parameter that can be used for the description of this criterion. In many animal welfare assessment protocols, however, body condition is included as an animal-based indicator to measure welfare. Both poor and excessive body conditions can lead to extreme and unexpected results. Poor body conditions can show signs of malnutrition, diseases, or extreme hunger. Poor and weak body conditions can have severe negative effects on the reproduction, behavior, and health of the animals [23]. 

To indicate the welfare problems, both poor and excessive body conditions can be used (indicator 1.1) [24]. To date, there is no scoring scale for the body condition of Punjab urial; hence, the body condition scoring guidelines for two other wild bovids proposed in [10,24] were followed. Animals were observed from a close distance from behind and the side. Punjab urial was scored as “poor body conditions” if ribs, spine, and pelvis were sharp and prominent, and projected with a concave rump. The animal was scored as “normal body condition” if ribs, pelvis, and spine were difficult to be distinguished from one another, with a flat rump area. The animal was scored as “excessive body conditions” if ribs, spine, and pelvis were not visible because of a thick layer of fat, with a protruding rump area.

#### 3.1.2. Lack of Prolonged Thirst 

One of the most important welfare requirements is ad libitum, access to water. Water provision in animal welfare protocol is a resource-based indicator. There should be easy access to water sources for the animals, containing clean and fresh water supplied on a daily basis.

According to the current study, water availability (indicator 2.1), in conjunction with the number of water sources (2.2) and cleanliness of water sources (2.3), should be properly checked. All these indicators were validated in [10,25] for assessing the welfare of captive *Dorcas gazelles* and cows. Water was scored as “dirty” if it was polluted with food wastes or plastics, or was dark green in color with a foul smell. On the contrary, water was scored as “clean” if it was without any contamination or foul smell. Remains of fresh feed were acceptable.

#### 3.1.3. Lack of Minerals 

In order to maintain normal physiological functions, wild ungulates usually search for a salt lick. Deficiency of salts can have severe negative effects on animal health, appetite, reproduction, and lactation [26]. Since captive animals are confined to limited areas in enclosures, they are more prone to mineral deficiency [27]. The current protocol included two indicators—(3.1) “availability of salt licks” and (3.2) “licking objects”—to check whether the animals were mineral-deficient. Salt-deficient individuals usually lick soil, woods, and even their own urine or that of other individuals [28]. According to the protocol for Punjab urial developed in this study, animals were scored as “mineral-deficient” if they showed any sign of licking objects or urine. 

#### 3.1.4. Thermal Ease

The natural habitat of the Punjab urial in Pakistan ranges from the dry, hot, and arid rocky mountains, to the high elevations in northern areas, which have extreme climatic conditions [29,30]. In captivity, there is a possibility of exposure for a long time to temperatures different from those of the natural habitat, which can eventually result in heatstroke and stress in animals [10]. In order to assess thermal ease, two indicators were developed: “shelter availability” (4.1) and “shade availability” (4.2). These indicators were assessed by checking whether all animals could simultaneously access shade during hot weather and shelter when climatic conditions are not favorable. These indicators were already validated in [10,19] for dorcas gazelles and domestic sheep.

#### 3.1.5. Easiness in Movement

Wild ungulates usually roam over vast areas, and their home ranges may exceed several square kilometers [31]. Wild animals kept in relatively small enclosures are more prone to develop different negative behavioral and physiological changes. All these negative developments are clear indicators of poor animal welfare [32]. According to [33], each Punjab urial should have a minimum area of 46.45 m^2^, and for every additional animal, this area should be increased by 25% (11.6 m^2^). Two indicators were developed in the current protocol, which had already been used and validated in [10,11,19]. The indicators included “total area of enclosure” (5.1), and “space (m^2^) offered per animal” (5.2), with the latter derived as the total enclosure area divided by the total number of animals in the area. 

#### 3.1.6. Standard Enclosures

The enclosures designed and constructed for breeding and conservation of wild animals should fulfill maximum criteria for standard enclosures. These criteria include fence structure and fixation to prevent the entry of other animals, especially small mammals. The fence should be covered so that animals do not have any visual contact with the outside environment if the aim is reintroduction. Each enclosure must have quarantine facilities, so that newly arrived animals can be held in isolation for observation, and sick or infected animals can be separated from other animals. Keeping in view the recommendations made in [34,35], four indicators, i.e., “fence structure” (6.1), “fence substratum” (6.2), “availability of quarantine” (6.3), and “number of quarantines” (6.4), were developed to assess enclosure standards. 

#### 3.1.7. Lack of Injuries

Due to limited space and the presence of aggressive animals, especially when different species are kept together, there is a chance of injuries [36]. Hoof injuries are common in artiodactylids [37]. There is a high chance of integument deformities and skin damage in captive animals. These deformities can result from any infectious disease, physical environment, aggression, or improper capture and handling of animals [10]. Two indicators were developed to assess lack of injuries: “integument deformities” (7.1) and “lameness” (7.2).

Integument deformities were assessed from close observation of the skin of animals. Hairless or damaged patches of skin of more than 2 cm were counted [10]. Animals were also observed for intraspecific aggression marks and injury marks from capture. Animals with “integument deformities” (7.1) were scored as follows: animals having several small lesions or a single lesion bigger than 2 cm^2^ and hairless patches were considered to have “severe integument deformities”; animals having no lesions, but having hairless patches were considered to have “moderate integument deformities”; and animals having no lesions or hairless patches were considered to be normal with “no integument deformities”.

All animals were observed for “lameness” (7.2) while they were moving. Animals having an apparent abnormality in walking or running were scored as “lame”, while those having no apparent abnormality were scored as “not lame”. The above indicators were already validated in [10,11,19]. 

#### 3.1.8. Lack of Disease

Common diseases found in Punjab urial (*Ovis vignei punjabiensis*) are viral infections, such as contagious ecthyma, and foot and mouth diseases (FMD). The major bacterial disease is pneumonia caused by *Pasteurella* spp. [17]. Other disorders that can affect the captive animals are behavioral disorders, birth problems, gastrointestinal infections, and trauma. Trauma mostly occurs due to intraspecific aggression, during capture and handling, or due to accident [10]. According to the American Association of Zoo Veterinarians [38], every facility holding wild animals, especially for conservation and breeding purposes, must have qualified veterinarians, a veterinary facility where sick animals can be treated, and a facility fornecropsy.

Six indicators were developed to assess the lack of diseases, including respiratory and gastrointestinal diseases, and health facilities. These indicators included “ophthalmic discharge” (8.1), “nasal discharge” (8.2), “labored breathing” (8.3), “diarrhea” (8.4), “availability of veterinarian” (8.5), and “availability of veterinary facility” (8.6). If any indicators among these were observed, animals were scored as “obvious”, and animals having no sign of these indicators were scored as “non-obvious”. All these indicators were validated in [25,27,39,40] for the welfare assessment of cattle. 

#### 3.1.9. Displaying Social Behavior

Agonistic behavior is displayed by animals usually in response to stress, intraspecific and interspecific competition for resources, and mating competition [41]. This behavior can result in severe physical damage. *Ovis vignei punjabiensis* is a gregarious ungulate with multiple agonistic and affinitive behaviors [22]. In captivity, no studies have been conducted on these animals to evaluate their social behavior. Extreme and repeated agonistic behavior displayed by animals in captivity can be a sign of poor management and welfare [10]. On the contrary, frequently occurring affinitive behavior represents satisfaction of animals concerning their environment, and hence good animal welfare. 

Two indicators were developed to assess the social behavior: “affinitive interactions” (9.1) and “agonistic interactions” (9.2). These indicators were also validated in [10,21,25]. An ethogram (Table 2) was developed based on the social behavior of Punjab urial [22], and information was obtained from a short reconnaissance survey for this study. 

#### 3.1.10. Group Dynamics

Punjab urial are gregarious ungulates [30] with different types of herds. Rams usually prefer to join herds in the rutting season; thus, the herd composition changes with the season. Three types of herds have been observed in wild Punjab urial: female herds exclusively comprising ewes and young; mixed herds containing one or more rams, ewes, and young; and male herds containing only males [22]. Husbandry guidelines suggest that in reproductive groups of captive ungulates, one adult male should be kept with 3–7 females and their young, while 3–7 adult males should be kept in bachelor groups [10]. In order to prevent inbreeding, reproductive males should be replaced or exchanged after every two years and females after three years [34].

Following [10], two indicators—“herd size” (10.1) and “herd composition” (10.2)—were developed to assess the group dynamics. Wildlife enclosures and zoos have a current tendency to build larger and more naturalistic facilities. Sometimes it is encouraged to mix species with other sympatric species where they share the same space and resources, which may serve as a source of enrichment, especially for animals raised for conservation and reintroduction purposes [41]. The current protocol included another indicator “number of animals (other species)” (10.3) [10], to record the number of individuals of other species. In addition to the number, any information about interspecific interactions based on the behavior should be recorded, which could be very useful for establishing management guidelines.

#### 3.1.11. Display of Other Behavior

Behavior that is invariable and repetitive without any apparent purpose is termed stereotypic behavior. Most of this behavior is due to stress, frustration, repeated attempts to cope, or failures of the central nervous system [27,42]. Sometime this behavior may help animals cope with hostile and difficult environments, but stereotypic behavior is generally regarded as an indicator of poor welfare [43]. There is no such information about stereotypic behavior in captive Punjab urial. Wild animals, especially ungulates in captivity, are more prone to develop oral stereotypies [44]. This protocol suggested that all animals should be checked for the presence or absence of “stereotypic behavior” (11.1). Animals should be scored as “stereotypes present” and “stereotypies absent” in the case of presence or absence of stereotypic behavior, respectively. Any behavior that is observed and recorded should be described properly [45]. The use of this indicator was strongly recommended in the welfare assessment captive animals and was validated successfully by [10].

Captive breeding programs can be promising for species that are raised for reintroduction, if environmental enrichment programs are encouraged. *Ovis vignei punjabiensis* prefers to live in rocky mountain areas with shrubs and grasses [29]. Although there are no husbandry guidelines for this species, guidelines for other species recommend providing opportunities for animals to perform their natural behaviors of browsing, grazing, running away, and hiding. In order to fulfill these requirements, it is recommended to make use of structural components like rocks, vegetation, and uneven ground, and to modify feeding techniques periodically with accompanying simulations of sounds and smell [10]. These practices will help produce captive-born animals suitable for release into the wild for reintroduction purposes. 

To assess “environmental enrichment programs” (11.2) [10], the animals’ access to opportunities such as browsing and grazing should be recorded. In addition, it should be taken into account whether certain objects, such as poles or sticks of different shapes and sizes, are used; if yes, then when and how they are used must be described. 

#### 3.1.12. Good Human–Animal Affiliations

For keeping captive populations healthy, it is recommended to minimize stress usually associated with veterinary procedures [37]. Arranging proper medical training programs under the supervision of experienced staff can help to reduce stress and injuries while handling and treating animals. In case no “medical training programs” (12.1) are arranged, then it is of utmost importance that the facility must hold advanced “capturing, handling, immobilization, and translocation” (12.2) systems that can ensure the minimization of stress, damage, or any loss of animals. These indicators were already validated for dorcas gazelles in [10].

### 3.2. Application of Punjab Urial Welfare Assessment Protocol

The following results were obtained after implementation of the proposed protocol.

#### 3.2.1. Lack of Prolonged Appetite 

According to the results obtained for body conditions (1.1) after application of the protocol, all animals had normal body condition with the exception of one adult male at CU1 with excessive body condition. According to the life history record of this male, it was never observed to have mated during the rutting season. 

#### 3.2.2. Lack of Prolonged Thirst 

The assessment of water sources (2.1) was conducted for all the three groups. At CU1, a concrete pond (0.91 × 0.91 m with a depth of 0.45 m) was present; CU2 had a concrete pond (1.52 × 1.52 m with a depth of 0.45 m) plus additional clay buckets (0.609 × 0.609 m); and CU3 had a concrete pond (2.74 × 2.74 m with a depth of 0.45 m) and three additional clay buckets (0.609 × 0.609 m). Water availability (2.2) and cleanliness of water sources (2.3) needed to be improved in CU2 and CU3. Both facilities had concrete ponds, but they were dry and the water in the clay buckets was not fresh and clean. Only the pond at CU1 had a sufficient supply of fresh water from a nearby spring. The study was conducted in the hot month of August and animals were frequently observed drinking water from this pond. 

#### 3.2.3. Lack of Minerals 

Salt licks were available (3.1) in sufficient quantity in each of the three facilities, and no animal was found to be licking objects (3.2). According to these results, animals showed no signs of mineral deficiency. 

#### 3.2.4. Thermal Ease 

Each of the three facilities provided shelters (4.1) for animals. At CU1, there were three shelters in total: two of 3.04 × 13.04 m (metal) and one of 3.96 × 3.04 m with a wooden roof. All animals were provided with enough shade (4.2) during the hot summer. In addition to the shelters, CU1 had many large sheesham (*Dalbergia sissoo*) trees providing thick cover for the animals. Animals were observed preferring to stay in the shade of trees rather than in artificial shelters. One shelter (15.24 × 6.09 m) was provided at CU2, and two shelters (15.24 × 6.09 m each) were provided at CU3. In addition, enclosures held enough vegetation (*Acacia modesta*) to provide shade for the animals. Every animal had access to shade and shelter in bad weather conditions (hot weather and rainy days).

#### 3.2.5. Easiness in Movement

The total area of the enclosures (5.1) and the space offered per animal (5.2) varied across the enclosures. Being constructed in or on the boundaries of wildlife parks, every enclosure encompassed a large area, with sufficient natural vegetation and uneven ground. CU1 was the only enclosure which lay close to local settlements and roads (Table 3). 

Although enough area was provided, sub enclosures should be designed and constructed within the main enclosures to separate different reproductive groups.

#### 3.2.6. Standard Enclosures

All three enclosures were assessed for existing fence conditions (6.1): whether it was covered or not, or broken. The fences in all the three enclosures were single layered and uncovered, but not broken at any point. The location of the enclosures and thick vegetation prevented the animals from eye contact with the public. In CU1, due to comparatively less vegetation and nearby road and settlements, the fence should be double wired and covered in order to avoid any physical or frequent eye contact with the public.

Fence substrata (6.2) were assessed and found to be in good condition, thus preventing the entry of other small mammals into the enclosures. None of the three enclosures had quarantine facilities (6.3 and 6.4). For robust captive breeding and management, the availability of quarantine facilities should be ensured.

#### 3.2.7. Lack of Injuries 

Animals were observed visually (also included the use of binoculars) for the assessment of integument deformities (7.1). No animal was found with any integument deformity. All animals were carefully assessed for lameness (7.2), but all the animals were normal. 

#### 3.2.8. Lack of Diseases 

Eight individuals (one adult male, three adult females, and four juveniles) at CU1 were observed to have ophthalmic discharge (8.1). None of the animals from CU1, CU2, or CU3 were observed to have signs of nasal discharge (8.2), labored breathing (8.3), or diarrhea (8.4). No classified veterinarian (8.5) or veterinary facility (8.6) was present at any of the three facilities. Veterinary records of each facility revealed that animal health checks were performed once a month, and the animals were checked by a classified veterinarian in case of mass infections. Keeping in view the budget constraints, optimum veterinary facilities in all the centers should be improved accordingly.

#### 3.2.9. Displaying Social Behavior

**Figure 1:**
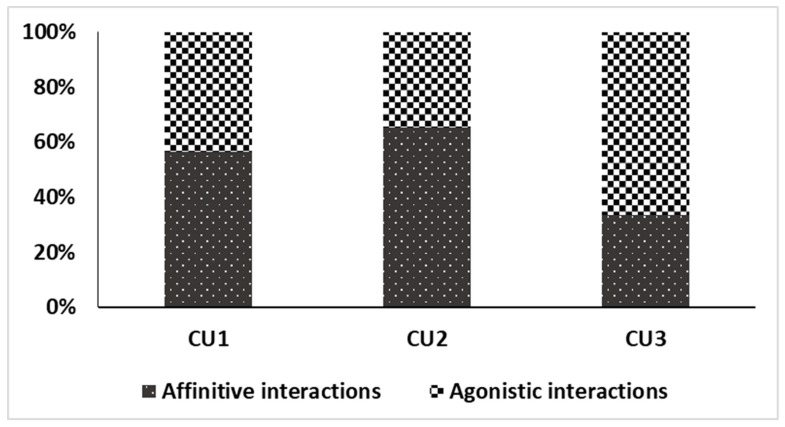
Percentage of affinitive and agonistic interactions of the total number of interactions recorded for each herd of Punjab urial assessed (*n* = 37).

Results for affinitive interactions (9.1) and agonistic interactions (9.2) showed that the highest aggression was shown at CU3 (66.59%), followed by CU1 (43.64%) and CU2 (37.75%) (Figure 1). The results obtained from the Shapiro–Wilk test (W = 0.634, *p* < 0.05) were significant. Thus, our data was not normally distributed. The Kruskal–Wallis H test showed that there was a statistically significant difference in behavior among the three groups (χ^2^ (2) = 10.073, *p* < 0.05), with a mean rank of 87.43 for CU1, 80.22 for CU2, and 109.00 for CU3. Animals at CU3 shared the space with other animals (Table 3).

While assessing the social behavior (9.1 and 9.2), the Punjab urial at CU3 showed aggression (chasing and head jerking) toward the chinkara. No obvious aggression was observed from Punjab urial toward mouflon sheep. All three species were observed maintaining approximately a distance of 10–20 m from each other.

#### 3.2.10. Group Dynamics

An assessment of the number of Punjab urial (10.1) in all the centers showed that only animals at CU3 were sharing space with other species, including chinkara (*Gazella bennettii*; *n* = 13) and mouflon sheep (*Ovis orientalis*; *n* = 3) (10.3). Results for assessing herd composition (10.3) were: CU1, *n* = 23 animals (adult male = 5, subadult male = 1, female = 6, young = 11); CU2, *n* = 6 (adult male = 3, adult female = 1, young = 2); and CU3, *n* = 8 (adult male = 4, adult female = 2, young = 2). Life history records of Punjab urial at each of the three facilities showed that no male had been replaced with other males from a different population. This situation was alarming and could potentially lead to inbreeding depression and drastic downfall of the population. These results strongly suggested that the exchange of productive males and females between different populations is necessary to avoid inbreeding.

#### 3.2.11. Display of Other Behavior

While assessing animals for stereotypic behavior (11.1), only two adult females and one sub-adult male at CU1 were found to move back and forth repetitively next to a fence and bite the fence. Regarding environmental enrichment (11.2), all the centers provided natural opportunities to forage, in the form of thick and tall vegetation for browsing. Due to comparatively fewer shrubs and less low vegetation at CU1, formal enrichment in the form of vertical poles or sticks to offer food should be encouraged. This will increase complexity in the physical environment of the enclosures.

#### 3.2.12. Good Human–Animal Affiliations 

Each of the herds assessed at the three different centers had never been a part of any medical program (12.1). Very poor results were recorded regarding capturing, handling, immobilization, and translocation techniques in all the three centers. Animal capture was mostly conducted by chasing animals and confining them to a corner of the enclosure. Veterinary and post-mortem reports revealed the expiry of seven chinkaras and three Punjab urials during capturing and translocation, respectively.

### 3.3. Overall Comparison 

Overall, all the three herds were same in terms of resource-based indicators. A slight difference among the three herds was present in terms of animal-based indicators. The herd at CU1 (43.64%) showed aggression and some animals (34.78%) were observed showing signs of ophthalmic discharge. The second herd showed an aggression of 37.75%, which was comparatively lower than that observed in the other two herds, but no diseased animal was found. The herd at CU3 showed the highest aggression (66.59%), but no animal was diseased. Comparing the results obtained for all the three groups, we assumed the herd at CU2 was at maximum welfare, followed by CU3 and CU1. 

## 4. Discussion 

The current project was designed to develop, for the first time in Pakistan, a baseline protocol to assess the welfare of wild ungulates in captivity. The assessment of resources, management, and animal-based measures are collectively termed as animal welfare assessments. In order to have a clear idea about the welfare of a group of animals in captivity, it is difficult to achieve the goals by having a single indicator or very few indicators. Thus, to have a complete and appropriate welfare assessment for a particular species, it is important to have a combination of several indicators according to the biology and ecology of that species [21]. The protocol proposed in this study is based on the welfare protocol for domestic sheep. This protocol differs from the domestic sheep welfare protocol in terms of the number of indicators, consisting of an extended list of 31 different animal- and resource-based indicators. In this newly developed protocol, we found that some indicators are difficult to assess with accurate results, especially in wild animals, when they are kept in large enclosures with dense vegetation. Although we used binoculars to assess integument and skin deformities (indicator 7.1), it was difficult to determine if there were any small lesions or patches on the body. According to [46], there is a high possibility of high levels of aggression and fights in wild ungulates in captivity. Detailed observations of skin and other integuments are thus very important. We do not encourage excluding this indicator; rather, we suggest the use of more powerful binoculars or a high-resolution camera to obtain clear pictures of the animals.

Our protocol is based on the welfare protocol for domestic sheep, but we excluded the criteria ‘positive emotional state’. There is a lack of information relative to this subject in captive ungulates [10]. This state includes pleasure, comfort, confidence, and interest. The aim of animal welfare assessment is to determine positive emotional states, or reduce undesirable experiences and increase opportunities for animals to have more healthy and positive states [47]. We suggest that these criteria should be included in developing welfare assessment protocols for any wild species in captivity. 

An emerging trend in establishing zoos and enclosures is to provide large and more natural environments for captive animals [48]. The current protocol found the area requirements for captive Punjab urial very true-to-life and acceptable in each of the three enclosures examined. All three enclosures offered vast areas to the animals, with natural habitats utilizing natural vegetation and uneven ground. All the animals could easily experience grazing, browsing, and athletic activities. With the exception of CU1, where animals were observed to be moving back and forth and the enclosure is located in close proximity to local settlements and roads, so stereotypic behavior was recorded during the application of the current protocol. According to [49], eye contact of visitors and wild animals in captivity can result in stress and stereotypic behavior. Following [34], fences at CUI should be covered with raffia in order to avoid frequent eye contact between the animals and the public.

Interspecific aggression has been mostly documented in carnivores [50], while such information for wild ungulates is scarce [51]. Interspecific aggression can possibly increase intraspecific aggression. In our study, we found that animals in CU3 showed the highest aggression (66.59%), followed by CU1 (43.64%) and CU2 (37.75%). We assumed that the higher aggression in CU3 was due to the presence of other species [52]. Punjab urial males were frequently observed chasing chinkara, showing comparatively less aggression toward mouflon sheep. We also recorded counter aggression from chinkara males toward Punjab urial and mouflon sheep. According to [36], interspecific aggression is usually greater between distantly related species than closely related species, and it is recommended to separate the species with aggressive males. Our results suggested isolating chinkara from Punjab urial. Regarding stereotypic behavior, it was observed only in three animals (two adult females and one subadult male) at CU1. This facility had less natural vegetation as compared to the other two facilities, where the animals did not show any stereotypic behavior. According to [45], captive ungulates have a high tendency to produce oral stereotypies when they have limited opportunities for natural foraging; those findings are in agreement with results produced from the current study. 

Recently, medical training programs and training techniques have been practiced and understood in modern zoos and facilities. These methodologies are frequently used and applied in different species, including big cats, elephants, giraffes, and apes. There is a lack of implementation of such programs in ungulates [10]. We consider it important to add a medical training program (12.2) because, if properly practiced, it is a promising means of reducing the stress caused by veterinary techniques. For capture of animals, every facility must have the right material (capture enclosure, net, handling crush, and dart gun), coupled with an experienced team, in order to avoid trauma and other serious injuries [34]. During application of the proposed protocol, it was found that several animals had expired during capture and translocation. These results make the medical training program a top priority in Punjab urial and other associated captive ungulates.

The development of this welfare assessment protocol is a leading documented work in developing a scientific and standard tool for the measurement of welfare in Punjab urial *(Ovis vignei punjabiensis*). Using the Welfare Quality^®^ protocol for farm animals as a reference, welfare assessment protocols for several wild species have been developed, including those for mink (*Neovison vison*), foxes (*Vulpes* spp.) [47], dorcas gazelles (*Gazella dorcas*) [10], and bottlenose dolphins (*Tursiops truncatus*) [47,53]. 

Welfare assessment protocols are developed with the aim of assessing the welfare level in captive animals, to discover limitations in captive breeding, and to ensure optimal welfare through recommendations. Protocols that are practical and easy to be applied are considered successful protocols. Protocols developed for different species differ in terms of time for their implementation. In the case of mink and foxes [47], the developed welfare assessment protocol needs three visits to each farm. In the case of bottlenose dolphins [53], the protocol requires two days for a complete welfare assessment of a dolphin pod including up to 10 individuals. Our protocol includes an extensive set of 31 indicators, and thus has some practical challenges. According to [10], protocols for dorcas gazelles require less than 6 h per herd. During the application of the proposed protocol, the largest herd of Punjab urial (*n* = 23) was assessed at CU1, and all the indicators were assessed in 5 h per herd. Our protocol is in early-stage development for assessing the welfare in captive Punjab urial and endorsement is still needed; however, its application to the three different herds and the results obtained allowed to identify some areas in all the facilities which need to be improved. 

## 5. Conclusions 

Using the welfare assessment protocol for farm animals (sheep) as the base, we developed a welfare protocol for welfare assessment in captive Punjab urial (*Ovis vignei punjabiensis*). This first specific protocol developed for Punjab urial comprised 4 basic principles, 12 criteria, and 31 animal- and environmental-based indicators. Although this protocol still needs validation, its first application and subsequent results obtained from three different herds of captive Punjab urial at CU1, CU2, and CU3 highlight some areas where improvements are essential. According to the results obtained from this protocol, handling, capturing, and translocation were found to be the most important areas for necessary action as most of the mortalities happened in capturing and translocation. Another important area which needs to be improved on an urgent basis is the availability of veterinary facilities. Furthermore, we recommend the shifting of breeding animals between different populations. Based on the results, we recommend covering the fence at CU1 in order to prevent frequent human–animal eye contact. On the basis of this study results, we believe that this protocol can be a promising tool for welfare assessment at facilities that hold Punjab urial. The current protocol has the best combination of welfare indicators for the target species and is a leading step in captive breeding research in Pakistan. In addition, this protocol can be used as a base for developing similar welfare protocols for other captive mountain ungulates in Pakistan and globally.

## Figures and Tables

**Table 1:** Punjab urial welfare quality assessment protocol principles, criteria, and indicators. Animal-based indicators are represented by *.

Welfare Principles	Criteria	Indicators
Good feeding	1. Lack of prolonged appetite	1.1 Body conditions *
	2. Lack of prolonged thirst	2.1 Number of water sources
		2.2 Water availability
		2.3 Cleanliness of water sources
	3. Lack of minerals	3.1 Availability of salt licks
		3.2 Licking objects
Good housing	4. Thermal ease	4.1 Shelter availability
		4.2 Shade availability
	5. Easiness in movement	5.1 Total area of enclosure
		5.2 Space (m^2^) offered per animal
	6. Standard enclosures	6.1 Fence conditions
		6.2 Fence substratum
		6.3 Availability of quarantine
		6.4 Number of quarantines
Good health	7. Lack of injuries	7.1 Integument deformities *
		7.2 Lameness *
	8. Lack of disease	8.1 Ophthalmic discharge *
		8.2 Nasal discharge *
		8.3 Labored breathing *
		8.4 Diarrhea *
		8.5 Availability of veterinarian
		8.6 Availability of veterinary facility
Appropriate behavior	9. Displaying social behavior	9.1 Affinitive interactions *
		9.2 Agonistic interactions *
	10. Group dynamics	10.1 Herd size
		10.2 Herd composition
		10.3 Number of animals (other species)
	11. Display of other behavior	11.1 Stereotypic behavior *
		11.2 Environmental enrichment programs
	12.Good human–animal affiliations	12.1 Medical training program
		12.2 Capturing, handling, immobilization, and translocation

**Table 2:** List of social behaviors included in the welfare protocol for Punjab urial.

Behavior Pattern	Description of Behavior
Mutual grooming	When an animal brushes another animal with its muzzle on any part of the body with exception to the anal region. If the actor animal stops brushing for 10 s and starts again, it is to be counted as a new bout, regardless of whether the actor brushes the same receiver or another. If the actor receives reversal brushing from the receiver, it should also be counted as a new bout (AFI).
Licking	One animal licks any part of another animal with the tongue with the exception of anal region or urine. If the actor animal stops licking for 10 s and starts again, it is to be counted as a new bout, regardless of whether the actor licks the same receiver or another. If the actor receives reversal brushing from the receiver, it should also be counted as a new bout (AFI).
Smelling	One animal smells any part of another animal with the exception of the anal region or urine (Flehmen response). If the actor animal stops smelling for 10 s and starts again, it is to be counted as a new bout, regardless of whether the actor smells the same receiver or another. If an actor receives reversal smelling from the receiver, it should also be counted as a new bout (AFI).
Play	Physical contact of two animals by rubbing bodies, horning, head to head play, or rubbing horns against the neck or other parts of the body, with no signs of aggression or taking advantage. If the actor stops for more than 10 s and then resumes with the same receiver or another, it should be counted as a new bout (AFI).
Chase	One animal running behind another animal (receiver), causing the receiver to flee from its previous position. The animal also shows strong aggression toward the receiver (AGI).
Block	One animal (actor) runs after another (receiver), stands broadly in front of the receiver, and prevents approaching the opposite sex (AGI).
Parallel walk	Two animals at the same time walking parallel with heads bent down and maintaining a distance of 10–20 m. If the animals scratch the ground with their feet, stop for 10 s or more, and resume, it is to be counted as a new bout (AGI).
Fighting and thrusting	Two animals raise their front legs and strike their heads and horns, or push one another back (head-to-head or horns’ base) planting legs on the ground with great force. One animal hits others with a kick or strong butting, or if any animal thrusts vegetation or another object with signs of aggression. If any animal displays such behavior and stops for 10 s or more and then resumes, it should be counted as a new bout (AGI).

AFI = Affinitive interactions, AGI = Agonistic interactions.

**Table 3:** Total enclosure area and area offered per animal in each herd, including individuals from other species (having the same approximate space requirements as Punjab urial).

Facility	Number of Animals	Total Area of Enclosure (Area) (m^2^)	Space Per Animal (m^2^/Animal)
CU1 (Cherat Wildlife Park)	23	14,299	621
CU2 (Manglot Wildlife Park)	6	3530	588
CU3 (Manglot Wildlife Park)	22 ^a^	15,793	717

^a^ Punjab urial, *n* = 8; chinkara, *n* = 13; mouflon sheep, *n* = 3.
